# Ophthalmolgy

**Published:** 1877-05-20

**Authors:** 


### OPHTHALMOLGY.

### MODIFIED OPERATION FOR CATARACT.

M. Galezowski, at the recent meeting of the French Association for the Advancement of Science, described an operation for cataract, which he had devised. At the present time, he said, "every surgeon endeavors to modify Graefe's operation, which is at present almost abandoned." He described the modifications he had adopted; he does not make the sclerotic puncture and counter puncture, but restricts the incision to the limits of the cornea; he abandons the linear method, and substitutes for it an incision at the lower portion of the cornea, making a small flap. He also excises the lower portion of the iris, though the deformity of the pupil is then more observable, but this, he thinks, will be but a slight inconvenience to those who desire one thing, the restoration of sight. M. G. attributes to this inferior excision, the great success he has obtained, which is at present 100 per cent. Of 67 operations performed in the city, he has not failed in a single instance. By his method, after the first steps of the operation the eye is free in its motions, especially after the removal of the lens, and to this M. G. attributes the rarity of the loss of the vitreous. Another modification, which he considers not less important, consists in the abandonment of the use of cystitome for the division of the capsule, and effecting this with Graefe's knife. As soon as the puncture is made the point of the knife is directed to the lens, and the capsule may be divided easily; after the counter puncture is effected the flap is made. Latterly, instead of excising the iris he has made a simple incision of the sphincter pupillæ with very satisfactory results, but he has not determined to adopt this definitely. The statistics given to show the value of his method were as follows: Of 385 operations, 67 were performed in private practice, of which last all were successful. Of 322 operated on at his clinic, 288 were successful.—*Revue Scientifique*, August 26, 1876.

